# Comparison of characteristic competencies of public health nurses working at a community general support center and health and welfare in public administration in Japan

**DOI:** 10.20407/fmj.2023-018

**Published:** 2024-05-29

**Authors:** Miho Miyamoto, Satoko Yanagisawa, Junko Fukada, Rumi Seko

**Affiliations:** 1 Faculty of Nursing, School of Health Sciences, Fujita Health University, Toyoake, Aichi, Japan; 2 School of Nursing & Health, Aichi Prefectural University, Nagoya, Aichi, Japan

**Keywords:** Community general support center, Public health nurse, Competency, Older adults, Preventive care

## Abstract

**Objectives:**

To compare the characteristic competencies of public health nurses working for the older adult’s health and welfare in public administration (“PA”) with those at community general support centers (“CGSC”) in Japan.

**Methods:**

We conducted a questionnaire survey by mail for PA and CGSC public health nurses. A competency list that was developed to compare three groups (PA, CGSC experts with ≥5 years of experience, and CGSC newcomers with ≤2 years of experience) was used. The following characteristics were examined: (1) competencies acquired early after arriving at the CGSC, (2) competencies acquired through a certain amount of CGSC experience, (3) common competencies, (4) competencies that even experts lacked, and (5) competencies that the newcomers lacked.

**Results:**

We examined the responses of 171 PA nurses, 185 CGSC expert public health nurses, and 165 CGSC newcomer public health nurses. The results of comparison of the three groups showed that (1) had no applicable items; (2) had nine items for individual support associated with preventive care management; (3) had 14 items including teamwork among three professionals (social workers, senior care manager, public health nurse)/other professionals and self-improvement; (4) had three items for community development, (5) had two items for individual support and 16 items for community development.

**Conclusion:**

Initiatives for preventive care and coordination of care teams should be supported and suggested as characteristic competencies for CGSC public health nurses.

## Introduction

In Japan, initiatives towards achieving a community general support care system have been taken considering problems such as increased social security costs and the seamless provision of medical and nursing care services due to the rapid decline in birthrate and aging of the population. Community general support centers (CGSCs) are core institutions that support older adults. They can be classified into two types: (1) directly-managed operated by municipalities; and (2) contracted type operated by private corporations. In Japan, there are 5,404 CGSCs (as on end of April 2022).^1^ CGSCs are staffed by three types of professionals (public health nurses, social workers, and senior care managers). The public health nurse is expected to a play role in enhancing the innate abilities of the older adults^2^ and spreading preventive care to the community.^3^

Public health nurses who have switched their career from the field of public administration health to work at CGSCs have mentioned that “differences in awareness have started emerging among the public health nurses who share the same values at health centers due to various affiliations”^4^ and “I sometimes lose sight of my role and do not know what to do”.^5^ The new organizational structure of the CGSCs and allocation of only one public health nurse to each center has increased occupational stress and decreased job satisfaction among the public health nurses working at these centers.^6^ Public health nurses with public administration experience working at CGSCs are often confused and unable to use their past experience; therefore, their behavior is believed to be different from that of other CGSC public health nurses. Currently, there is no specific education or training program for becoming a CGSC public health nurse; despite the lack of ability and skills among these nurses, there is a lack of indicators to determine the required learning.

Recently, the concept of competency^7^ has been introduced in human resource development, and research in the field of public health nursing is progressing domestically and internationally.^8–10^ Competency is defined as the attitude, knowledge, skills, thinking, and behavioral characteristics that are fundamentally inherent in people who deliver high performance at their jobs.^7^ Previous research has reported competency scales such as those that cover activities of public health nurses,^11^ and public health nurses in the field of public administration^12,13^ and at the managerial level.^14^ In the field of research on older adults, reports have been published on activity indicators for public health nurses who promote preventive care systems^15^ and perspectives on the activities of CGSC public health nurses^16–18^; however, there is no research that focuses on the competencies of CGSC public health nurses.

Therefore, the purpose of this study was to clarify the characteristic competencies of CGSC public health nurses by comparing them with those of public health nurses belonging to the field of health and welfare in public administration (“PA public health nurses”) for older adults. “Competency” in the present study refers to the behavior of an outstanding CGSC public health nurse who provides high-quality support to older adults. “High-quality support to older adults” refers to support for old or frail old adults that helps them lead an independent and positive life among family, neighbors, professionals, and community residents; this individual support helps create an organic system that enables them to lead a peaceful life in old age as well.

## Methods

### Creation of question items

The question items were based on the CGSC public health nurse competency list^19^ created in previous research. This list consisted of 80 items in five areas: “individual support”, “community development”, “teamwork among three professionals at a CGSC, “self-improvement”, and “job management”. The “individual support” section consisted of 30 items including actions that provide individual support to older adults, such as assessments that critically evaluate the lives of older adults, actions that build relationships of trust that allow for the expression of true feelings, communication that increases motivation, and actions for coordinating the care team that supports older adults. The “community development” section consisted of 22 items including actions evaluating the current situation of the community by assessment of daily tasks, and those promoting community development with the aim of nursing care prevention, along with the residents. The “teamwork among three professionals at a CGSC” section consisted of 11 items including actions for improving relationships among the three occupations and contribution with one’s own expertise. The “self-improvement” section consisted of 13 items including the acquisition of specialized knowledge and techniques as well as actions to improve expertise. The “job management” section consisted of four items including prompt responses required in emergencies and actions for conducting tasks in a planned manner while taking priorities into consideration. The evaluation method involved evaluation of the question “are you able to perform the behavior specified in the question item?” with a five-point Likert scale of 5=very well, 4=somewhat able, 3=cannot say either way, 2=somewhat unable, and 1=completely unable. The higher the score, the higher was the competency.

A pilot survey was conducted from June to August 2017 to confirm whether the respondents on this list would interpret and respond to each item using the constructed concepts. Requests were made to 50% of the 2,495 randomly selected CGSCs across Japan, and the questionnaires were sent after consent was obtained from 831 people at 513 facilities. Responses were obtained from 505 people (response rate of 60.8%), and we analyzed 419 people (valid response rate of 83.0%) who responded to the complete questionnaire. We conducted exploratory factor analysis and deleted the first item that had similar loadings on multiple factors. Additionally, there seemed to be a variation in the perception of the fifth item amongst the respondents; therefore, the language of the fifth item was revised to add a scenario in which the behavior occurs. Cronbach’s α coefficient for the entire list after one item was deleted was 0.97. As a result, we used a list of five areas, 20 categories, and 79 items as the final version. It should be noted that staff structures differ between PA and CGSCs; therefore, the term “three...professionals” in “teamwork among three professionals at a CGSC” and the text of “social workers and senior care managers” were revised to read “colleagues and other professionals” in the question item for PA public health nurses.

### Research subjects

We targeted public health nurses belonging to CGSCs and PAs.

We randomly selected CGSCs nationwide, excluding those targeted in the pilot survey, at a rate of 50% and stratified them into directly managed and contracted types. Then, for the public health nurses affiliated with each CGSC, we selected those with CGSC experience of ≥5 years and those with CGSC experience of ≤2 years as subjects.

Meanwhile, for PA public health nurses, we targeted public health nurses who were affiliated with municipalities covering a population of ≥20,000 people, excluding municipalities with directly managed systems, and those who had ≥5 years of experience as a public health nurse and ≥2 years of experience of being in charge of older adults. We then requested up to two people per facility for responses. However, we excluded those who had experience working at a CGSC.

### Data collection method

A self-administered questionnaire survey was conducted by mail from October to December 2017. The evaluation method was the same as that used in the pilot study. Public health nurses belonging to PAs and CGSCs have different duties and roles; therefore, this list may have included items that are not the duties of PA public health nurses or items that they have no opportunity at all to perform. Therefore, we also asked PA public health nurses the following question, “in your work over the past year, did you have the opportunity to practice the behaviors shown in the competency list”, and we added “0: no opportunity to practice it”.

The basic questions posed for the public health nurses included those regarding age, sex, and number of years of experience as a public health nurse. Furthermore, for CGSC public health nurses, the facility type and number of years of experience in a CGSC were also enquired.

### Analysis method

In the analysis, for each item, “5: Able to do so very well” was given 5 points, “4: Somewhat able to do so” was given 4 points, “3: Cannot say either way” was given 3 points, “2: Somewhat unable to do so” was given 2 points, and “1: Unable to do so at all” was given 1 point. For “0: No opportunity to practice it” was interpreted as an inability to do the activity due to lack of opportunity, and this was scored as 1 point, which was the same as “1: Unable to do so at all”. Additionally, we calculated the average value for each of the 79 items and those within each area as the total score and area score, respectively.

Next, we divided the subjects into the following three groups in order to clarify the characteristic competencies of CGSC public health nurses: a) PA public health nurses, b) experienced CGSC public health nurses with ≥5 years of experience in CGSCs (“CGSC experienced public health nurses”), and c) newcomer CGSC public health nurses with ≤2 years of experience in CGSCs (“CGSC newcomer public health nurses”). Comparative analysis was conducted for these three groups using the following process for total score, five area scores, and list items. Results for confirming the distributions of the total score, five area scores, and each item score on the list for normality using the Shapiro-Wilk test showed that the total score and the three area scores of “individual support”, “community development”, and “self-improvement” of only the CGSC newcomer public health nurses exhibited normality; however, none of the other groups exhibited normality. Therefore, analysis was conducted using the Kruskal-Wallis test, and multiple comparisons were conducted using Dunn’s method. Comparisons between the three groups were judged using the following criteria (1)–(5). (1) The competency that could be acquired early after arriving at the CGSC was significantly higher for CGSC experienced public health nurses and CGSC newcomer public health nurses than that for PA public health nurses (i.e., a<b, and a<c). (2) The competency that could be acquired through a certain amount of experience at a CGSC was significantly higher for CGSC experienced public health nurses than that for PA public health nurses and CGSC newcomer public health nurses (i.e., a<b and c<b). (3) The competency common to both PA and CGSC public health nurses was high in both PA public health nurses and CGSC public health nurses (i.e., a>3.8 and b>3.8). The reason for setting 3.8 as the benchmark was that this value exceeded the average values for PA public health nurses and CGSC experienced public health nurses and was around +1 SD of CGSC newcomer public health nurses, based on the average value and standard deviation of the total score. (4) The competency that not even CGSC experienced public health nurses had yet acquired was low for both CGSC newcomer public health nurses and CGSC experienced public health nurses (i.e., b<3.0 and c<3.0). The reason for setting 3.0 as the benchmark was that a score of ≤2 was a negative response, with “2: Somewhat unable to do so” and “1: Unable to do so at all”. The competency that was lacking in CGSC newcomer public health nurses was low in CGSC newcomer public health nurses (i.e., c<3.0). IBM SPSS Statistics ver. 24 Windows version was used for the analysis, with a significance level set at 5%.

### Ethical considerations

We provided written explanations to research subjects and facility managers stating the following: purpose and aims of this study, voluntary co-operation of the participants, anonymity of the survey, and provision of consent by filling out and returning the questionnaire. The research request form described the research purpose and methods, protection of personal information, voluntary research cooperation, and publication of research results. This study was conducted with the approval of the Aichi Prefectural University research ethics review committee (approval number: 29APUI No. 11-5).

## Results

There were 2,448 targeted CGSCs, comprising 600 directly-managed facilities (24.5%) and 1,848 contracted facilities (75.1%). The research request forms were mailed to the facility managers. Questionnaires were sent to 876 public health nurses at 559 facilities who had provided consent, and 567 responses (64.7%) were received. We excluded 94 people with missing values, for a total of 473 subjects (valid response rate of 83.4%). From this, 185 people with CGSC experience of ≥5 years and 165 people with CGSC experience of ≤2 years were analyzed.

In case of PA public health nurses, we contacted the older adult support sections in 760 municipalities, estimating two subjects per facility, for a total of 1,520 subjects, and received responses from 217 people (valid response rate of 14.3%). We excluded 43 people with missing values and three people with <5 years of public health nurse experience, and analyzed 171 people (valid response rate of 78.8%).

### Overview of subjects

Table 1 shows the basic attributes of the three subject groups.

### Characteristic competencies of CGSC public health nurses

Table 2 shows the results of the Kruskal-Wallis test for the total score and area score. “Individual support” was (2) the competency acquired through a certain amount of experience. “Community development” was (5) the competency that CGSC newcomer public health nurses lacked.

The supplementary table shows the results of the Kruskal-Wallis test for each item. There was no item for (1) the competency that could be acquired early after arriving at the CGSC. The (2) items that could be acquired through a certain amount of experience were the nine “individual support” items. The (3) items that were common between the public health nurses belonging to PAs and CGSCs were one “individual support” item, seven “teamwork among three professionals at a CGSC”, five “self-improvement” items, and one “job management” item. The (4) items that could not yet be sufficiently acquired even by CGSC experienced public health nurses were three “community development” items. The (5) items lacking in CGSC newcomer public health nurses were two “individual support” items and 16 “community development” items.

## Discussion

### Characteristic competencies of CGSC public health nurses

A comparison of the three groups in the five areas showed that “individual support” was significantly higher in CGSC experienced public health nurses than in PA public health nurses. This area is thought to be a characteristic competency for CGSC public health nurses and will be discussed in detail below.

The results for each item showed that there were no items judged as a (1) competency that could be acquired early after arriving at the CGSC. Yoshida et al.^15^ reported that the awareness of the importance of understandings needs was higher among those with ≤5 years of experience as it is a basic activity that is practiced from the start of arriving at the CGSC. In the present study, we asked whether or not the subjects were able to take action in a practical situation; therefore, the subjects did not reach a point of self-evaluation that they were able to act as such at the two-year mark.

The (2) competencies that were significantly higher in the CGSC experienced public health nurses than in the PA public health nurses were the nine “individual support” items, including life assessment skills to support older adults to prepare for end-of-life situations based on their living conditions, ability to make a proposal to increase older adults’ efforts to engage in preventive care, and ability to coordinate and enhance collaboration in the care team for individual support. However, “No opportunity to practice it” accounted for 7.0–19.9% of the responses for these nine items among PA public health nurses, and it is thought that the competency of PA public health nurses was underestimated. However, public health nurses who responded with “No opportunity to practice it” may not have necessarily had significantly high abilities.

Hino^20^ stated that the competencies required for CGSC staff include being knowledgeable about a variety of topics, such as the daily lives of community residents, and the ability to build networks, gather information, and take proactive action. In the results of the present study as well, competencies that were higher in CGSC experienced public health nurses than in PA public health nurses included the following: list No. 4, which is the act of going to the location to confirm the actual living conditions by integrating objective information and proposing a hypothesis, to provide a reasonable explanation to older adults of the incomprehensive situation; list No. 18, which is the act of promptly responding to general consultations and requests from residents and related parties; and list No. 21, which is the act of communicating on a regular basis when engaging with team members or welfare workers who provide individual support. Providing higher quality support by using information and networks of community life that have been cultivated through many individual support cases is thought to require a certain amount of practice.

Additionally, list No. 30, which included items like the act of collecting information on the flow of use of service offices and residential facilities, status of the facilities, and whether emergency response was possible, had a significantly higher value for CGSC experienced public health nurses than that for PA public health nurses. Older adults may require immediate nursing care in case of sudden worsening of their condition or decline in their activities for daily living; therefore, information on nursing care services and facilities need to be collected and prepared on a regular basis. Considering that CGSCs function as a general consultation center for older adults, the ability to take action for collecting community information is thought to be a competency that is required in practice. When comparing the duties of a PA public health nurse to the core duties of one at CGSC and to the clerical duties related to long-term care insurance, the functions of the ones at CGSC, who directly responds to consultations from older adults, differ, and there are few opportunities for practice. Therefore, it is inferred that there are also few opportunities for PA public health nurses to increase their competencies. Considering all of these factors, the nine “individual support” items are thought to be characteristic competencies for CGSC public health nurses.

“Community development” was the competency with the lowest score in all three groups; however, the results showed that it was particularly low among newcomer public health nurses. Additionally, the competencies that could not be sufficiently acquired even by CGSC experienced public health nurses were three “community development” items, which included list No. 44 (conduct business using the subsidies from national and local governments or the important tasks of the affiliated facility); and list No. 52 (communicate the effect of the business implemented to the residents). Previous research has also indicated that the need of community development is understood; however, concrete efforts have not yet been taken.^21^ The “community development” competency was not sufficiently acquired not only by CGSC newcomer public health nurses but also by CGSC experienced public health nurses, suggesting the need for the development of a training program.

It could be inferred from the results that showed a significant difference between CGSC experienced public health nurses and newcomer public health nurses that competency is acquired in stages. It is thought that training programs tailored to each stage should be provided: during the newcomer stage, training should be provided to enhance preventive care management skills, such as individual support for older adults and their families and coordination of care teams. Furthermore, at the stage where the nurse has obtained a certain amount of experience, training should be provided for the commercialization of community-building activities, work evaluation, and feedback to residents.

The (3) competencies that were common to both PA public health nurses and CGSC experienced public health nurses were the following items: “teamwork among three professionals at a CGSC, “self-improvement”, and “job management”. It is thought that collaborating with colleagues and other professionals, managing work for efficient proceedings, and self-improvement of the necessary knowledge and skills are common competencies for public health nurses.

### Limitations of this study

This study has the following limitations. First, the results of this study cannot be generalized as the subjects of the present study were only a small portion of the public health nurses nationwide. Additionally, the subjects who cooperated in this study may have been highly interested people. Second, the validity of the list that was used in the present study was verified through consultation with an expert panel. This is because CGSC public health nurses include those who are transferred within a short period of time, so there are few experts, and their identification is also difficult, which precluded the use of a large-scale consensus building method. However, in future, if CGSCs become more specialized, and the number of public health nurse experts increases, then it may be possible to conduct content validity verification on a larger scale, using the Delphi method. Additionally, this list was based on qualitative research and expert judgment in order to establish theoretical fields. However, this study revealed that competencies between CGSC experienced public health nurses and PA public health nurses could be differentiated. Hence, it is thought that confirmatory factor analysis could be conducted in future in order to set fields that correspond to response trends, as long as none of the items are dropped.

## Figures and Tables

**Table1:** Subject attributes of the three groups

	Item	a		b		c	
		PA public health nurse n=171		CGSC experienced public health nurse n=185		CGSC newcomer public health nurse n=165	
		n	%	n	%	n	%
Gender	Male	8	4.7	5	2.7	6	3.6
	Female	163	95.3	180	97.3	159	96.4
Age	20s	3	1.8	2	1.1	36	21.8
	30s	42	24.6	41	22.2	63	38.2
	40s	75	43.9	70	37.8	39	23.6
	50s	47	27.2	60	32.4	22	13.3
	60s	4	2.3	12	6.5	5	3.0
	70s	0	0.0	0	0.0	0	0.0
	Mean±Standard variation	44.7±7.7		46.7±8.4		38.4±9.7	
Years of experience as a health nurse	Under 2 years	0	0.0	0	0.0	66	40.0
	2–4	0	0.0	0	0.0	14	8.5
	5–9	23	13.5	35	18.9	25	15.2
	10–14	24	14.0	37	20.0	15	9.1
	15–19	36	21.1	27	14.6	14	8.5
	20–24	39	22.8	31	16.8	15	9.1
	25–29	27	15.8	27	14.6	6	3.6
	30–34	15	8.8	14	7.6	5	3.0
	35 or more years	7	4.1	14	7.6	5	3.0
	Mean±Standard variation	20.1±8.0		19.1±9.7		9.4±10.3	
Facility management	Direct management	—		100	54.1	62	37.6
	Commissioned management			85	45.9	103	62.4

**Table2:** Comparison between the three groups for CGSC public health nurse competency list total score and five area scores n=521

Domain		n	Mean	Standard variation	“0: No opportunity to practice”^d^		*p*				Determination^g^
					Average number of respondents	Average%	group comparison^e^	multiple comparisons^f^			
Total points	a	171	3.49	0.59	12.2	7.1		a–b	0.13		
	b	185	3.63	0.38			<0.01	a–c	0.02	c<a	
	c	165	3.36	0.49				b–c	<0.01	c<b	
Individual support	a	171	3.44	0.83	18.6	10.9		a–b	<0.01	a<b	
	b	185	3.75	0.36			<0.01	a–c	0.12		②
	c	165	3.46	0.48				b–c	<0.01	c<b	
Community development	a	171	3.21	0.82	17.0	9.9		a–b	1.00		
	b	185	3.21	0.63			<0.01	a–c	<0.01	c<a	⑤
	c	165	2.80	0.73				b–c	<0.01	c<b	
Teamwork among three professionals at a CGSC	a	171	3.88	0.55	1.8	1.1		a–b	0.05		
	b	185	4.00	0.57			0.01	a–c	1.00		③
	c	165	3.87	0.65				b–c	0.02	c<b	
Self-improvement	a	171	3.74	0.53	0.3	0.2		a–b			
	b	185	3.70	0.45			0.15	a–c			
	c	165	3.65	0.50				b–c			
Job management	a	171	3.66	0.61	2.5	1.5		a–b	0.35		
	b	185	3.77	0.53			0.02	a–c	0.15		
	c	165	3.53	0.62				b–c	<0.01	c<b	

a: PA public health nurse b: CGSC experienced public health nurse c: CGSC newcomer public health nurse. d: Average number and percentage of people who responded with “0: No opportunity to practice it”, which was an option only for PA public health nurses e: Kruskal-Wallis test, f: Dunn test. g: The circled numbers in the judgment indicate (2): competency acquired through certain amount of experience at CGSC, (3) common competency, and (5) competency lacking in CGSC newcomer public health nurses.

## Supplementary Material

PDF-Japanese
