# Supplementary material for: Comparison of characteristic competencies of public health nurses working at a community general support center and health and welfare in public administration in Japan
Source: Fujita Med J. 2024 May 29;10(3):75–80. doi: 10.20407/fmj.2023-018 (PMC11288718; doi:10.20407/fmj.2023-018)
Supplement: Supplementary file 1 — PDF-Japanese [file fmj-10-075-s001.pdf]

Abstract

**目的：**行政高齢者保健福祉分野（以下，行政）保健師と比較し，地域包括支援センター（以下，地域包括）保健師に特徴的なコンピテンシーを明らかにする．

**方法：**行政と地域包括の保健師に郵送による質問紙調査を行った．研究者らが開発したコンピテンシーリストを用い，行政，地域包括で経験 5 年以上のベテランと 2 年以下の新人の 3 群を比較し，①地域包括着任後早期に獲得するコンピテンシー，②一定の地域包括の経験を経て獲得されるコンピテンシー，③共通コンピテンシー，④ベテランでも不足しているコンピテンシー，⑤新人に不足しているコンピテンシーを検討した．

**結果：** 行政 171 人，地域包括のベテラン 185 人と新人 165 人を分析対象とした．3 群比較の結果，①は該当項目がなかった．②は介護予防ケアマネジメントに関連する個別支援 9 項目，③は 3 職種（社会福祉士，主任介護支援専門員，保健師）/他職種のチームワーク，自己研鑽等 14 項目，④は地域づくり 3 項目，⑤は個別支援 2 項目，地域づくり 16 項目が抽出された．

**結論：**地域包括保健師に特徴的なコンピテンシーとして，介護予防につながる取組みへの支援とケアチームのコーディネートが示唆された．

## 1．序論

わが国では，急激な少子高齢化の進展に伴い社会保障費の増加や医療・介護の切れ目ないサービス提供等の課題を背景に，地域包括ケアシステムの実現に向けた取組みが進められている．地域包括支援センター(Community general support center, CGSC)は，高齢者の生活を支える中核機関で，市町村が運営する直営型と民間法人に業務委託している委託型があり，全国 5,404 箇所（2022 年 4 月末現在）に設置されている [1)]．地域包括支援センターには，保健師，社会福祉士，主任介護支援専門員の 3 職種が配置され，保健師の役割として，高齢者の本来持っている力を高める役割 [2)]や，介護予防を地域に広める役割 [3)]等が期待されている．

行政保健分野から地域包括支援センターに異動した保健師より，「保健センターで価値観を共有していた保健師との間に所属の違いによる意識の差が生じるようになった [4)]」，「自分の役割を見失いどうしていいかわからないことがある [5)]」などの言葉が聞かれている．また，地域包括支援センターという新たな組織体制や保健師一人配置が多いという職場環境において，地域包括支援センターで働く保健師の職業性ストレスや職務満足感低下が懸念されている [6)]．行政経験のある保健師が地域包括支援センターに配属された際，これまでの経験が応用できず戸惑う状況があることから，地域包括支援センター保健師に必要とされる行政保健師とは異なる行動特性があるのではないかと考えられた．しかし，地域包括支援センター保健師に特化した教育や研修はなく，能力やスキルが不足していると認識しながらも何を学修すればよいかを判断する指標がないという現状である．

近年人材育成においてコンピテンシーの概念 [7)] が導入され，国内外の公衆衛生看護領域で

もコンピテンシーの研究が進められている[8-10]．コンピテンシーとは，職務に対し高い業績を上げている人に根源的に備わっている考え方や態度，知識やスキル，行動特性とされている[7]．先行研究では，保健師活動の一部の活動領域を取り上げたコンピテンシー[11]や，行政分野に所属する保健師[12-13]，管理職レベルの保健師に求められるコンピテンシー[14]等の尺度が報告されている．高齢者分野では，介護予防システムを推進する保健師の活動指標[15]や，地域包括支援センター保健師の活動視点などが報告されている[16-18]が，地域包括支援センター保健師のコンピテンシーに着目した研究はみられない．

そこで本研究の目的は，地域包括支援センター保健師に特徴的なコンピテンシーを，行政保健福祉分野の高齢者担当課に所属している保健師（以下、行政保健師）との比較において明らかにすることである．本研究において「コンピンテンシー」とは，質の高い高齢者支援を実践する卓越した地域包括支援センター保健師の行動とした．「質の高い高齢者支援」とは，高齢者あるいは虚弱高齢者が，家族，近隣住民，専門職の支援や見守りを得ながら，自立的に，前向きに生活できるように支援すること，およびその個別支援を基盤として，高齢者を含む地域住民が，高齢になっても安心して生活できる有機的な仕組みをつくることとした．

## 2. 研究方法

1）*質問項目の作成*

質問項目は，先行研究にて作成した地域包括支援センター保健師のコンピテンシーリスト[19]を原案とした．本リストは，「個別支援」「地域づくり」「地域包括支援センター3 職種のチ

ームワーク」「自己研鑽」「業務マネジメント」の 5 領域 80 項目で構成される．「個別支援」は，高齢者を個別支援する行動で，高齢者の生活を批判的にみるアセスメントや本心を語ってもらえるような信頼関係を構築する行動，意欲を高めるコミュニケーション，高齢者を支援するケアチームを調整する行動など 30 項目で構成されている．「地域づくり」は，日常業務から地域の現状をアセスメントし，住民と共に介護予防を目指した地域づくりを進める行動など 22 項目で構成される．「地域包括支援センター3 職種のチームワーク」は，3 職種間の関係を良好にする，自身の専門性で貢献する行動など 11 項目で，「自己研鑽」は，専門知識や技術の習得，専門性を高める行動など 13 項目で構成される．「業務マネジメント」は，緊急時に求められる迅速な対応や優先順位を考え計画的に業務を遂行する行動など 4 項目で構成されている．評価方法は，「質問項目の行動を求められた際，実行できるか否か」を 5＝非常によくできる，4＝だいたいできる，3＝どちらともいえない，2=あまりできない，1＝全くできないの 5 件法のリッカートスケールで評価し，得点が高いほどコンピテンシーが高くなるように設定した．

本リストの回答者が，各項目に対して構成された概念で解釈し回答されるかを確認するためにパイロット調査を 2017 年 6〜8 月に行った．対象者は，全国の地域包括支援センターの 50%を無作為抽出した 2,495 施設に依頼し，513 施設 831 人より承諾が得られ調査票を発送した．505 人（回収率 60.8%）から回答を得，完全回答であった 419 人（有効回答率 83.0%）を分析対象とした．探索的因子分析を行い，複数の因子に類似の負荷量をもつ 1 項目を削除し，5 項目は，回答者によって質問文に示された行動の捉え方が異なっていることが推測さ

れたため，行動する場面を追記する文言修正を行った．1 項目削除したリスト全体のクロンバックのα係数は 0.97 であった．結果，5 領域，20 カテゴリー， 79 項目のリストを完成版として用いた．なお，行政高齢者保健福祉分野と地域包括支援センターとは，職員体制が異なるため,「地域包括支援センター3 職種のチームワーク」の項目の“3 職種”や“社会福祉士や主任ケアマネージャー”の文言を行政保健師への質問項目には“同僚や他職種”に修正した.

2）*研究対象者*

地域包括支援センター保健師および行政保健師とした.

地域包括支援センターは，50%の割合で無作為抽出したパイロット調査で対象とした施設を除いた全国の地域包括支援センターを直営型と委託型に層化し，各地域包括支援センターに所属する保健師で, 地域包括支援センター経験 5 年以上および 2 年以下の者を対象とした.

一方，行政保健師は，直営型を設置している市区町村を除く全国の人口 2 万人以上の市区町村に所属している保健師で，保健師経験が 5 年以上かつ高齢者分野を 2 年以上担当している者を対象とし，1 施設 2 名まで回答を求めた．ただし，地域包括支援センター勤務経験のある者を除いた.

3）*データ収集方法*

2017 年 10～12 月に郵送による自記式質問紙調査を行った．評価方法は，パイロット調査と同様とした．行政保健師と地域包括支援センター保健師とは，業務や役割が異なるため，本リストには，行政保健師の業務ではない項目や全く実施する機会のない項目が含まれる可

能性がある．そのため，行政保健師には，「ここ 1 年間の業務の中で，コンピテンシーリストで示す行動について実践する機会があったか否か」についても合わせて質問し「0：実践する機会がない」を追加した．

保健師の基本属性は，年齢，性別，保健師経験年数を尋ね，地域包括支援センター保健師にはさらに，施設形態と地域包括支援センター経験年数を尋ねた．

4）*分析方法*

分析は，各項目について「5：非常によくできる」は 5 点，「4：だいたいできる」は 4 点，「3：どちらともいえない」は 3 点，「2：あまりできない」は 2 点，「1：全くできない」は 1 点とした．「0：実践する機会がない」については，機会が無いために行動できないと捉え「1：全くできない」と同点の 1 点として得点化した．また，総得点および領域得点として各々79 項目，領域内項目における平均値を求めた．

次に，地域包括支援センター保健師に特徴的なコンピテンシーを明らかにするため，対象者を以下の 3 群に分けて検討した．a 行政保健師，b 地域包括支援センター経験 5 年以上のベテラン地域包括支援センター保健師（以下，地域包括ベテラン保健師），c 地域包括支援センター経験 2 年以下の新人地域包括支援センター保健師（以下，地域包括新人保健師）の 3 群について，総得点，5 領域得点およびリスト項目ごとに下記のプロセスで比較分析をした．総得点，5 領域得点およびリスト各項目得点の分布を Shapiro-Wilk 検定により正規性を確認したところ，地域包括新人保健師のみの総得点および「個別支援」「地域づくり」「自己研鑽」の 3 つの領域得点では正規性が確認されたが，それ以外は正規性が認められなかったため，

Kruskal-Wallis 検定を用いて分析し，Dunn の方法を用いて多重比較を行った．3 群の比較は以下の①～⑤の基準で判定した．①地域包括支援センターに着任後，早期に獲得可能なコンピテンシーとして，地域包括ベテラン保健師および地域包括新人保健師の方が行政保健師より有意に高い，つまり $a<b$ かつ $a<c$ とした．②地域包括支援センターでの一定の経験を経て獲得されるコンピテンシーとして，地域包括ベテラン保健師の得点が行政保健師および地域包括新人保健師よりも有意に高い，つまり $a<b$ かつ $c<b$ とした．③行政および地域包括支援センターのどちらの保健師にも共通するコンピテンシーとして，行政保健師および地域包括ベテラン保健師共に得点が高い，つまり $a>3.8$ かつ $b>3.8$ とした．3.8 と設定した理由は，総得点の平均値および標準偏差より，行政保健師，地域包括ベテラン保健師の平均を超え，かつ地域包括新人保健師の+1SD 付近として設定した．④地域包括ベテラン保健師であってもまだ十分に獲得できていないコンピテンシーとして，地域包括新人保健師かつベテラン保健師の得点が低い，つまり $b<3.0$ かつ $c<3.0$ とした．3.0 と設定した理由は，得点 2 点以下は「2 : あまりできない」,「1 : 全くできない」の否定的な回答であるため，3.0 未満と設定した．⑤地域包括新人保健師に不足しているコンピテンシーとして，地域包括新人保健師の得点が低い，つまり $c<3.0$ とした．解析には IBM SPSS Statistics ver.24 Windows 版を用いて分析し，有意水準は 5%とした．

5）*倫理的配慮*

　研究対象者および施設管理者に文書で，本研究の主旨と目的および協力するか否かは自由意志であり，本調査は無記名とし，質問紙への記入・返送をもって同意とすることを説明し

た．研究依頼書に研究目的と方法，個人情報保護，自由意志による研究協力，研究成果の公表について記載した．なお，本研究は愛知県立大学研究倫理審査委員会の承認を得て実施した（承認番号：29 愛県大学情第 11-5 号）．

## 3. 研究結果

対象とした地域包括支援センターは 2,448 施設で, 直営型 600 施設 (24.5%), 委託型 1,848 施設（75.1%）へ研究依頼書を施設管理者に郵送した．同意が得られた 559 施設の保健師 876 人に調査票を送付し，567 人（回収率 64.7%）から回収した．欠損値があった 94 人を除外した 473 人（有効回答率 83.4%）から，地域包括支援センター経験 5 年以上の 185 人，2 年以下の 165 人を分析対象とした.

行政保健師は，760 か所の市区町村高齢者支援担当課へ 1 施設あたり対象者 2 人と推定し 1,520 人を対象として，217 人（回収率 14.3%）から回収した．欠損値があった 43 人および保健師経験 5 年未満であった 3 人を除外し, 171 人 (有効回答率 78.8%) を分析対象とした.

1）*対象者の概要*

対象者 3 群の基本属性を表 1 に示す.

2）*地域包括支援センター保健師に特徴的なコンピテンシー*

総得点および領域得点に対する Kruskal-Wallis 検定の結果を表 2 に示す.「個別支援」は，②一定の経験を経て獲得されるコンピテンシーであった．「地域づくり」は，⑤地域包括新人保健師に不足しているコンピテンシーであった.

各項目の Kruskal-Wallis 検定の結果を Supplementary table に示す．①着任後早期に獲得が可能なコンピテンシー項目はなかった．②一定の経験を経て獲得される項目は，「個別支援」の 9 項目であった．③行政保健師と地域包括ベテラン保健師とに共通する項目は，「個別支援」の 1 項目，「地域包括支援センター3 職種のチームワーク」の 7 項目，「自己研鑽」の 5 項目，「業務マネジメント」の 1 項目であった．④地域包括ベテラン保健師であってもまだ十分に獲得できていない項目は，「地域づくり」の 3 項目であった．⑤地域包括新人保健師に不足している項目は，「個別支援」の 2 項目と「地域づくり」の 16 項目であった．

**4．考察**

1） *地域包括支援センター保健師に特徴的なコンピテンシー*

5 領域の 3 群比較では，「個別支援」が，行政保健師よりも地域包括ベテラン保健師の方が有意に高く，この領域は地域包括支援センター保健師に特徴的なコンピテンシーであると考えられ，後述にて詳しく考察する．

次に，項目ごとの結果について，①着任後早期に獲得可能なコンピテンシーとして判定された項目はなかった．吉田ら[15)]は，ニーズ把握は新任期から実践する基本的活動であるため，ニーズ把握の重要性の認識は，経験 5 年未満の方が高かったと報告している．本研究では，実践場面で行動ができるか否かを質問したため，行動できると自己評価できるところまでは 2 年では至っていなかったと考えられる．

②の行政保健師よりも地域包括ベテラン保健師の方が有意に高かったコンピテンシーは，「個別支援」の 9 項目で，【高齢者の生活実態に基づいて，人生最期の場面に向けて送りたい

生活を支援する】ための生活アセスメント力,【高齢者の介護予防への取組み意欲が高まるような提案をする】力,【個別支援のためのケアチームの連携が高まるようにコーディネートする】力などであった．しかし，これら 9 項目の行政保健師の選択肢「実践する機会がない」の回答が 7.0～19.9％あり, 行政保健師のコンピテンシーが過小評価された可能性が考えられる．しかし,「実践する機会がない」と回答した保健師は，これらのコンピテンシーを維持・向上させる機会をもっていない者であり，その能力が著しく高いということは考えにくい.

日野[20]は，地域包括支援センター職員に必要な力量として，地域住民の日常等さまざまなものを知り得ておく必要があり，人脈づくりや情報収集力，積極的な行動力を述べている.本結果においても，地域包括ベテラン保健師が行政保健師よりも高かったコンピテンシーには，リスト No.4 の高齢者の不可解な状況に対して合理的な説明ができるように，客観的な情報を統合して仮説を立てて現場に出向き，実際の生活状況を確認する行動やリスト No.18 の住民や関係者からの総合相談や依頼に対してすぐ対応する行動，リスト No.21 の個別支援をしているチームメンバーの関係者や民生委員などと会った時に普段からコミュニケーションをとる行動が含まれていた．多くの個別支援の事例を通して培った地域の生活の情報やネットワークを生かしてより質の高い支援を行うことは，一定の実践の積み重ねが必要であると考えられる.

また, リスト No. 30 の緊急対応ができるように, サービス事業所や入所施設の利用の流れ,施設の状況，緊急対応が可能か否かを情報収集しておく行動は，行政保健師よりも地域包括ベテラン保健師の方が有意に高かった．高齢者は病状が急に悪化したり ADL が低下したり

すると，すぐに介護が必要となるため，普段から介護サービスや施設の情報を収集し備えておく必要がある．地域包括支援センターは高齢者総合相談窓口である施設の機能からも，地域の情報を収集する行動力は，実践で求められるコンピテンシーであると考えられる．行政保健師の業務は，地域包括支援センターの基幹的な業務や介護保険に関する事務的な業務など，高齢者の相談に直接対応する地域包括支援センターと機能が異なり実践の機会が少ないため，行政保健師のコンピテンシーが高められる機会も少ないと推察する．これらを総合して考えると，「個別支援」の 9 項目は，地域包括支援センター保健師に特徴的なコンピテンシーであると考えられる．

「地域づくり」は，3 群のいずれも最も得点が低かったコンピテンシーであるが，特に新人保健師で低いことが明らかになった．また，地域包括ベテラン保健師であっても十分に獲得できていないコンピテンシーは，「地域づくり」の 3 項目で，リスト No. 44 の国や市町村の補助金や所属施設の重点課題を利用して事業を行う，リスト No .52 の実践している事業の効果を，住民に伝える行動などであった．先行研究でも，地域づくりの必要性は理解しつつも具体的な取り組みに至っていないこと [21] が報告されている．「地域づくり」のコンピテンシーは，地域包括新人保健師だけでなくベテラン保健師も十分獲得されていない状況であり，研修プログラムの開発が必要であることが示唆される．

地域包括ベテラン保健師と新人保健師との間で有意な差が認められた結果より，コンピテンシーの獲得には段階があることが推察される．新任期には，高齢者と家族への個別支援およびケアチームの連携等の介護予防ケアマネジメント技術を高める研修を，また一定の経験

を経た段階では，地域づくりに向けた活動の事業化や事業評価，住民へのフィードバックに関する研修など，段階に合わせた研修プログラムの検討が必要と考えられる．

③行政保健師と地域包括ベテラン保健師ともに共通するコンピテンシーは,「地域包括支援センター3 職種のチームワーク」および「自己研鑽」,「業務マネジメント」の項目であった．同僚や他職種と協働することや業務を効果的に進められるようマネジメントすること，必要な知識や技術を自己研鑽することは, 保健師に共通するコンピテンシーであると考えられる．

2）*本研究の限界*

本研究には，以下の限界がある．第 1 に，本研究対象者は，全国の保健師の一部にとどまっていることから研究結果の一般化には注意が必要である．また本研究に協力した対象者は,関心の高い者であった可能性があることが考えられる．第 2 に，本研究で使用したリストの妥当性は，専門家パネルによる協議にて検証したものである．これは，地域包括支援センター保健師は, 短期間で異動する者もいるため, 専門家の人数が少なくまた特定が困難であり，大規模なコンセンサス形成手法を用いることができなかったためである．しかし， 今後地域包括支援センターの専門性が高まり，保健師専門家が増加した場合は，Delphi 法を含めたより大規模な内容妥当性の検証が可能だと思われる．また本リストは，質的研究と専門家の判断に基づく理論的な分野設定を行っているが，今回，地域包括支援センターベテラン保健師と行政保健師とのコンピテンシーの弁別が可能である項目が明らかになったことから，今後確証的因子分析を行って，項目が脱落しない範囲で，回答傾向に呼応した分野設定を試みることも可能だと考える．

**謝辞**

本研究にご協力くださいました全国の地域包括支援センターおよび行政高齢者保健福祉分野の保健師の皆様に深謝いたします．また，本研究を進めるにあたりご指導を賜りました元愛知県立大学看護学部教授の岡本和士先生に心から感謝を申し上げます．本研究は，JSPS 科研費 17K12552 の助成を受けて実施した．

**利益相反**

開示すべき COI はない．

【文献】

1. Health, Labour and Welfare Statistics Association. Kokumineisei no doukou (National health trends). Tokyo: Health, Labour and Welfare Statics Association; 2023. 238 (in Japanese).
2. Okamoto R. Chiikihokatsusiensenta ni kakawaru hokenshi no yakuwari (Role of public health nurses involved in community general support centers). The Japanese Journal for Public Health Nurse 2009; 65: 26-30 (in Japanese).
3. Kiyota K. Chokueishiki no chiikihokatsushiensenta to kaigoyobokatsudo no genzai (Directly operated community general support centers and current care prevention activities). The Japanese Journal for Public Health Nurse 2006; 62: 922-9 (in

Japanese).

4. Yumoto T, Murakami K. Hokenshi ha “chiikihokatsu kea” ni do mukiau ka (How do public health nurses deal with “comprehensive community care”). The Japanese Journal for Public Health Nurse 2005; 61: 1190-4 (in Japanese).
5. Kawahara M, Sugita K, Kodama C, Ono M. Chiikihokatsushiensenta no kino kyoka ni kakawaru hokenshi no katsudo jittai to kadai (The present state and challenges of public health nursing relating to the improvement of the function of community general support centers). Miyazaki Prefectural Nursing University Research and Training Center annual reports 2014; 3: 33-42 (in Japanese).
6. Sakurai M, Saeki K, Takahashi Y, Shido K, Kanbara R, Otomo Y, Nagai M, Miyake H. Hokkaido no chiikihokatsushiencenta ni kinmusuru hokenshi no shokugyosei sutoresu to shokumu manzokudo (Occupational stress and job satisfaction of public health nurses working at Community General Support Centers in Hokkaido). Hokkaido Journal of Public Health 2011; 24: 49-56 (in Japanese).
7. Spencer LM, Spencer SM. Competence at work : Models for Superior Performance. New York: Wiley; 1993: 11-9.
8. Chiikihoken jujisha no shishithu no kojo ni kansuru kentokai (Study group on improving the quality of community health workers). Chiikihoken o sasaeru jinzai no ikusei (Developing human resources to support community health). Tokyo: Chuohoki

Publishing; 2004: 69-75 (in Japanese).

9. Koshu eisei kango no arikata ni kansuru kentokai (Study committee on the state of publis health nursing). Koshueisei Kango no arikata ni kansuru kentoiinkai katsudohokoku "Hokenshi no koa karikyuramu ni tsuite" chukanhokoku (Report on the activities of the study committee on the state of public health nursing "On the core curriculum for public health nurses"). Japanese Journal of Public Health 2005; 52(8): 758-64 (in Japanese).

10. Quad Council of Public Health Nursing Organization. Public health nursing competencies. Public Health Nurs 2004; 21 : 443-52.

11. Shiomi M, Okamoto R, Iwamoto S. Development of competency measurement concerning the creation of projects/ social resources for public health nurses: Investigation of reliability and validity. Japanese Journal of Public Health 2009; 56: 391-401 (in Japanese).

12. Iwamoto S, Okamoto R, Shiomi M, Development and evaluation of the reliability and validity of a scale for basic actions relevant to public health. Japanese Journal of Public Health 2008; 55: 629-39 (in Japanese).

13. Saeki K, Izumi H, Uza M, Takasaki H. Development of a Way to Measure the Practical Competence of Public Health Nurses. Journal of Japan Academy of Community Health Nursing 2003; 6: 32-9 (in Japanese).

14. Hatono Y, Suzuki H, Masaki N. Development of the role scale for municipal supervising public health nurses. Japanese Journal of Public Health 2013; 60: 275-84 (in Japanese).

15. Yoshida R, Izumi H, Katakura Y, Namikawa K. The Development of Guidelines in Public Health Nursing Practice for the Promotion of Preventive Long-term Care Systems. Journal of Japan Academy of Community Health Nursing 2012; 14: 5-13 (in Japanese).

16. Kawamoto A, Taguchi A, Kuwahara Y, Matsunaga A, Iwasaki R, Murashima S. Contents of Individual Support Implemented by Cooperation between Neighbors and Public Health Nurses at Comprehensive Community Support Centers in Japan. Journal of Japan Academy of Community Health Nursing 2012; 15: 109-18 (in Japanese).

17. Ohtaka N, Sasaki A, Tanuma T, Morita K. A view of public health nurses working Community General Support Center on care prevention in community activities for elder people. Journal of the Ochanomizu Association for Academic Nursing. 2012; 6: 70-80 (in Japanese).

18. Yoshida R, Izumi H, Namikawa K. Public health nursing practice in the promotion of long-term care prevention systems with a focus on collaboration between community residents and public health nurses. Bulletin of Social Medicine. 2011; 28(1): 65-73 (in

Japanese).

19. Miyamoto M, Yanagisawa S. Competencies of public health nurses who work at a Community general support center. Journal of Japan Academy of Nursing for Home Care 2023; 11: 57-67 (in Japanese).

20. Hino N. Essential Abilities for the Staff of a Regional Comprehensive Support Center . Journal of Japan Academy of Home Care 2018; 22: 131-41(in Japanese).

21. Shirai K, Sugiura K, Tsushita K. Self-evaluation and worries of regional inclusive elderly-support centers and expected roles of prefectures. Japanese Journal of Public Health 2017; 64: 630-7 (in Japanese).

Table 1. Subject attributes of the three groups

| | | a | | b | | c | |
|---|---|---|---|---|---|---|---|
| | | PA public health nurse | | CGSC experienced public health nurse | | CGSC newcomer public health nurse | |
| | | n＝171 | | n＝185 | | n＝165 | |
| | Item | n | % | n | % | n | % |
| Gender | Male | 8 | 4.7 | 5 | 2.7 | 6 | 3.6 |
| | Female | 163 | 95.3 | 180 | 97.3 | 159 | 96.4 |
| Age | 20s | 3 | 1.8 | 2 | 1.1 | 36 | 21.8 |
| | 30s | 42 | 24.6 | 41 | 22.2 | 63 | 38.2 |
| | 40s | 75 | 43.9 | 70 | 37.8 | 39 | 23.6 |
| | 50s | 47 | 27.2 | 60 | 32.4 | 22 | 13.3 |
| | 60s | 4 | 2.3 | 12 | 6.5 | 5 | 3.0 |
| | 70s | 0 | 0.0 | 0 | 0.0 | 0 | 0.0 |
| | Mean±Standard variation | 44.7±7.7 | | 46.7±8.4 | | 38.4±9.7 | |
| Years of experience as a health nurse | Under 2 years | 0 | 0.0 | 0 | 0.0 | 66 | 40.0 |
| | 2 - 4 | 0 | 0.0 | 0 | 0.0 | 14 | 8.5 |
| | 5 - 9 | 23 | 13.5 | 35 | 18.9 | 25 | 15.2 |
| | 10 - 14 | 24 | 14.0 | 37 | 20.0 | 15 | 9.1 |
| | 15 - 19 | 36 | 21.1 | 27 | 14.6 | 14 | 8.5 |
| | 20 - 24 | 39 | 22.8 | 31 | 16.8 | 15 | 9.1 |
| | 25 - 29 | 27 | 15.8 | 27 | 14.6 | 6 | 3.6 |
| | 30 - 34 | 15 | 8.8 | 14 | 7.6 | 5 | 3.0 |
| | 35 or more years | 7 | 4.1 | 14 | 7.6 | 5 | 3.0 |
| | Mean±Standard variation | 20.1±8.0 | | 19.1±9.7 | | 9.4±10.3 | |
| Facility management | Direct management | — | | 100 | 54.1 | 62 | 37.6 |
| | Commissioned management | | | 85 | 45.9 | 103 | 62.4 |

Table 2 Comparison between the three groups for CGSC public health nurse competency list total score and five area scores n=521

| Domain | | n | Mean | Standard variation | "0: No opportunity to practice"[d] | | *p* | | | | Determination[g] |
|---|---|---|---|---|---|---|---|---|---|---|---|
| | | | | | Average number of respondents | Average% | group comparison[e] | multiple comparisons[f] | | | |
| Total points | a | 171 | 3.49 | 0.59 | 12.2 | 7.1 | | a-b | 0.13 | | |
| | b | 185 | 3.63 | 0.38 | | | <0.01 | a-c | 0.02 | c<a | |
| | c | 165 | 3.36 | 0.49 | | | | b-c | <0.01 | c<b | |
| Individual support | a | 171 | 3.44 | 0.83 | 18.6 | 10.9 | | a-b | <0.01 | a<b | |
| | b | 185 | 3.75 | 0.36 | | | <0.01 | a-c | 0.12 | | ② |
| | c | 165 | 3.46 | 0.48 | | | | b-c | <0.01 | c<b | |
| Community development | a | 171 | 3.21 | 0.82 | 17.0 | 9.9 | | a-b | 1.00 | | |
| | b | 185 | 3.21 | 0.63 | | | <0.01 | a-c | <0.01 | c<a | ⑤ |
| | c | 165 | 2.80 | 0.73 | | | | b-c | <0.01 | c<b | |
| Teamwork among three professionals at a CGSC | a | 171 | 3.88 | 0.55 | 1.8 | 1.1 | | a-b | 0.05 | | |
| | b | 185 | 4.00 | 0.57 | | | 0.01 | a-c | 1.00 | | ③ |
| | c | 165 | 3.87 | 0.65 | | | | b-c | 0.02 | c<b | |
| Self-improvement | a | 171 | 3.74 | 0.53 | 0.3 | 0.2 | | a-b | | | |
| | b | 185 | 3.70 | 0.45 | | | 0.15 | a-c | | | |
| | c | 165 | 3.65 | 0.50 | | | | b-c | | | |
| Job management | a | 171 | 3.66 | 0.61 | 2.5 | 1.5 | | a-b | 0.35 | | |
| | b | 185 | 3.77 | 0.53 | | | 0.02 | a-c | 0.15 | | |
| | c | 165 | 3.53 | 0.62 | | | | b-c | <0.01 | c<b | |

a: PA public health nurse b: CGSC experienced public health nurse c: CGSC newcomer public health nurse.

d: Average number and percentage of people who responded with "0: No opportunity to practice it", which was an option only for PA public health nurses

e: Kruskal-Wallis test, f: Donn test

g: The circled numbers in the judgment indicate (2): competency acquired through certain amount of experience at CGSC, (3) common competency, and (5) competency lacking in CGSC newcomer public health nurses.
