# Supplementary material for: Comparison of characteristic competencies of public health nurses working at a community general support center and health and welfare in public administration in Japan
Source: Fujita Med J. 2024 May 29;10(3):75–80. doi: 10.20407/fmj.2023-018 (PMC11288718; doi:10.20407/fmj.2023-018)
Supplement: Supplementary file 2 — Supplementary Table [file fmj-10-075-s002.pdf]

Supplementary table. Comparison between three groups of 79 items in competency list for CGSC public health nurses n=521

Area	Category	No.	List		n	Average value	Standard deviation	"0: No opportunity to practice"[d] Number of respondents	"0: No opportunity to practice"[d] %	*p* group comparison[e]	*p* multiple comparisons[f]			Determination[g]
Individual support	Support older adults to prepare for end-of-life situations based on their living conditions.	1	Collect and assess information from a wide viewpoint including medical care, living conditions, economic conditions, sense of value, living history, family relationship, community life, etc. of the older adult.	a	171	3.71	0.92	12	7.0		a-b	0.13		
				b	185	3.95	0.45			<0.01	a-c	0.02	c<a	
				c	165	3.65	0.61				b-c	<0.01	c<b	
		2	Specifically see and listen to the living activities of the older adult to understand their actual living and foresee future risks.	a	171	3.68	0.94	14	8.2		a-b	0.01	a<b	
				b	185	3.98	0.38			<0.01	a-c	0.17		②
				c	165	3.68	0.59				b-c	<0.01	c<b	
		3	Assess fundamental issues without confusing the demand and needs of the older adult.	a	171	3.52	0.95	13	7.6		a-b	0.37		
				b	185	3.74	0.56			<0.01	a-c	<0.01	c<a	
				c	165	3.38	0.64				b-c	<0.01	c<b	
		4	Go to the location to confirm the actual living conditions by integrating objective information and proposing a hypothesis, to provide a reasonable explanation to older adults of the incomprehensive situation.	a	171	3.36	1.07	20	11.7		a-b	<0.01	a<b	
				b	185	3.77	0.57			<0.01	a-c	0.82		②
				c	165	3.33	0.73				b-c	<0.01	c<b	
		5	Recognize small changes in the older adult such as facial expressions and tone of voice.	a	171	3.64	0.98	14	8.2		a-b	0.06	a<b	
				b	185	3.95	0.58			0.04	a-c	1.00		
				c	165	3.78	0.66				b-c	0.14		
		6	Assess the older adult from the viewpoint of care prevention.	a	171	3.69	0.92	12	7.0		a-b	0.01	a<b	
				b	185	4.00	0.49			<0.01	a-c	0.51		②
				c	165	3.71	0.62				b-c	<0.01	c<b	
		7	Listen to the honest intentions and assertions of the older adult such as what kind of living or life they would like.	a	171	3.62	1.05	17	9.9		a-b	0.03	a<b	
				b	185	3.96	0.58			0.02	a-c	1.00		
				c	165	3.81	0.63				b-c	0.10		
	Develop a kind of relationship for the older adult to speak their true feelings.	8	Create a relaxing atmosphere and take time to listen to allow the older adult to speak their true feelings, keeping in mind that they tend to refrain from speaking feelings of withdrawal or making requests.	a	171	3.63	1.03	15	8.8		a-b	0.04	a<b	
				b	185	3.95	0.57			0.02	a-c	1.00		
				c	165	3.76	0.72				b-c	0.07		
		9	Develop a relationship to be trusted as a public health nurse by providing proper information at a proper time based on individuality.	a	171	3.53	1.05	17	9.9		a-b	0.16		
				b	185	3.81	0.58			<0.01	a-c	0.07		
				c	165	3.46	0.77				b-c	<0.01	c<b	
		10	Develop a relationship with the older adult according to their sense of value and level.	a	171	3.63	1.01	15	8.8		a-b	0.01	a<b	
				b	185	3.97	0.45			<0.01	a-c	1.00		②
				c	165	3.76	0.60				b-c	<0.01	c<b	
	Make a proposal to increase older adults' efforts to engage in preventive care.	11	Propose a support method to draw out the strength of the older adult and enhance willingness to make efforts.	a	171	3.42	1.04	17	9.9		a-b	0.57		
				b	185	3.66	0.66			0.01	a-c	0.17		
				c	165	3.38	0.76				b-c	<0.01	c<b	
		12	Make multiple proposals about support methods to meet the needs of the older adult for them to choose from.	a	171	3.42	1.08	19	11.1		a-b	<0.01	a<b	
				b	185	3.90	0.57			<0.01	a-c	1.00		②
				c	165	3.50	0.77				b-c	<0.01	c<b	
		13	Continue to wait until the older adult become motivated after making proposals while accepting their feelings.	a	171	3.15	1.09	19	11.1		a-b	<0.01	a<b	
				b	185	3.61	0.65			<0.01	a-c	0.14		
				c	165	3.45	0.71				b-c	0.17		
	Increase family functions.	14	Respect the living, thoughts and ideas of the family.	a	171	3.81	0.93	12	7.0		a-b			
				b	185	4.05	0.45			0.15	a-c			③
				c	165	3.96	0.51				b-c			
		15	Make proposals that can be mutually compromised in order to coordinate between the older adult and family.	a	171	3.56	1.06	18	10.5		a-b	0.33		
				b	185	3.83	0.55			0.02	a-c	0.70		
				c	165	3.59	0.70				b-c	0.02	c<b	
		16	Include the family when considering methods to support the older adult.	a	171	3.69	1.04	16	9.4		a-b			
				b	185	3.86	0.58			0.17	a-c			
				c	165	3.75	0.58				b-c			
		17	If there are other issues in the family in the areas of mother-child, adult issues and mental issues, refer to a proper consultation institution to increase family functions.	a	171	3.78	0.99	14	8.2		a-b	1.00		
				b	185	3.92	0.61			<0.01	a-c	<0.01	c<a	
				c	165	3.58	0.86				b-c	<0.01	c<b	
	Obtain trust from related parties, welfare workers, etc. who come to be newly involved through individual support.	18	Promptly respond to general consultation and requests from residents and related parties.	a	171	3.73	0.99	12	7.0		a-b	<0.01	a<b	
				b	185	4.12	0.62			<0.01	a-c	1.00		②
				c	165	3.88	0.71				b-c	<0.01	c<b	
		19	If it is not possible to promptly respond to individual cases of general consultation, frequently follow up with progress to display the attitude of continuous involvement to residents and related parties.	a	171	3.44	1.04	15	8.8		a-b	0.01	a<b	
				b	185	3.79	0.62			0.01	a-c	0.99		
				c	165	3.65	0.70				b-c	0.16		
		20	Attend community events and meetings to get to know the residents and interact with them.	a	171	3.04	1.25	29	17.0		a-b	0.01	a<b	
				b	185	3.50	0.96			0.01	a-c	0.36		
				c	165	3.33	1.03				b-c	0.37		
	Coordinate and enhance collaboration in the care team for individual support.	21	Communicate on a regular basis when engaging with team members or welfare workers who provide individual support.	a	171	3.61	1.11	21	12.3		a-b	<0.01	a<b	
				b	185	4.07	0.58			<0.01	a-c	1.00		②
				c	165	3.76	0.79				b-c	<0.01	c<b	
		22	Reflect on support and confirm the results of activities with the related parties of team members or welfare workers who are providing individual support.	a	171	3.01	1.14	25	14.6		a-b	0.12		
				b	185	3.31	0.78			0.03	a-c	1.00		
				c	165	3.06	0.87				b-c	0.05		
		23	Consult with public heath nurses in other areas including the administrative area with respect to the method of individual support.	a	171	3.37	1.16	17	9.9		a-b	1.00		
				b	185	3.39	0.92			<0.01	a-c	<0.01	c<a	
				c	165	3.05	1.05				b-c	0.01	c<b	
		24	Create an opportunity for the related parties and welfare workers who provide individual support to meet face-to-face for discussion.	a	171	3.22	1.19	24	14.0		a-b	0.28		
				b	185	3.51	0.80			<0.01	a-c	0.02	c<a	
				c	165	3.02	0.95				b-c	<0.01	c<b	
		25	Share with the team members who provide individual support about the fact of going toward the same goal.	a	171	3.45	1.09	21	12.3		a-b	0.36		
				b	185	3.72	0.67			<0.01	a-c	0.06		
				c	165	3.38	0.84				b-c	<0.01	c<b	
		26	Persistently look for someone who might be able to support the older adult and introduce them.	a	171	2.96	1.15	25	14.6		a-b	0.34		
				b	185	3.19	0.82			0.01	a-c	0.53		
				c	165	2.87	0.93				b-c	0.01	c<b	⑤
		27	Meet and request the related parties and neighbors to pay attention and help, so that individual support can be continued when the public health nurse is absent.	a	171	2.89	1.22	34	19.9		a-b	<0.01	a<b	
				b	185	3.42	0.76			<0.01	a-c	1.00		②
				c	165	3.00	0.86				b-c	<0.01	c<b	
		28	Coordinate the team by understanding each role and duty of related parties of team members and welfare workers who provide individual support and by considering how they can function well.	a	171	3.19	1.13	22	12.9		a-b	0.15		
				b	185	3.50	0.72			<0.01	a-c	0.03	c<a	
				c	165	3.04	0.88				b-c	<0.01	c<b	
		29	Imagine the case when the condition of the older adult deteriorates and plan on how to respond to coordinate among related institutions.	a	171	3.39	1.12	22	12.9		a-b	0.99		
				b	185	3.62	0.67			<0.01	a-c	<0.01	c<a	
				c	165	3.19	0.85				b-c	<0.01	c<b	
		30	Collect information on the flow of use of service offices and residential facilities, status of the facilities, and whether emergency response was possible.	a	171	3.12	1.18	26	15.2		a-b	<0.01	a<b	
				b	185	3.57	0.74			<0.01	a-c	0.03	c<a	②
				c	165	2.96	0.93				b-c	<0.01	c<b	⑤
Community development	Understand the tasks in the community based on requests from residents obtained from individual support or care prevention classes as well as on local conditions.	31	Understand characteristics, awareness, etc. of the older adult and residents in the community in relation to general consultation or health education such as care prevention.	a	171	3.45	1.01	14	8.2		a-b	0.15		
				b	185	3.71	0.63			<0.01	a-c	0.05		
				c	165	3.35	0.76				b-c	<0.01	c<b	
		32	Foresee community issues and methods to solve issues by accumulating opinions from and information on residents obtained from contacting them through general consultation or health education such as care prevention.	a	171	3.37	1.02	14	8.2		a-b	1.00		
				b	185	3.51	0.64			0.01	a-c	0.03	c<a	
				c	165	3.24	0.75				b-c	0.01	c<b	
		33	Clarify what is predicted as issues by public health nurses through opinion exchange with residents and related parties.	a	171	3.08	1.09	18	10.5		a-b	0.89		
				b	185	3.25	0.75			<0.01	a-c	0.02	c<a	
				c	165	2.83	0.94				b-c	<0.01	c<b	⑤

		34	Consider care prevention to support the overall community residents, not only the older adult.	a	171	3.36	1.08	15	8.8		a-b	1.00		
				b	185	3.48	0.77			<0.01	a-c	<0.01	c<a	
				c	165	3.08	0.90				b-c	<0.01	c<b	
	Have a specific achievable vision based on community assessment.	35	Foresee the future community to explain the vision of how it should be.	a	171	3.36	1.00	8	4.7		a-b	1.00		
				b	185	3.31	0.84			<0.01	a-c	<0.01	c<a	
				c	165	2.81	0.98				b-c	<0.01	c<b	⑤
		36	Envision a method that could be implemented in the region in charge to accomplish the vision.	a	171	3.15	1.06	14	8.2		a-b	1.00		
				b	185	3.20	0.85			<0.01	a-c	<0.01	c<a	
				c	165	2.69	0.87				b-c	<0.01	c<b	⑤
	Explain to community residents.	37	Present data with evidence and explain community issues to the residents to arouse their problem consciousness.	a	171	3.19	1.08	18	10.5		a-b	1.00		
				b	185	3.17	0.93			<0.01	a-c	<0.01	c<a	
				c	165	2.61	0.98				b-c	<0.01	c<b	⑤
		38	Explain community issues to the residents by utilizing opportunities of every community gathering to share community issues with them.	a	171	2.94	1.20	27	15.8		a-b	1.00		
				b	185	3.04	0.92			<0.01	a-c	<0.01	c<a	
				c	165	2.58	0.92				b-c	<0.01	c<b	⑤
	Move the residents by utilizing the network cultivated from daily tasks.	39	Assess awareness of residents toward community development to plan for the timing to proceed with projects.	a	171	2.88	1.17	28	16.4		a-b	1.00		
				b	185	2.99	0.92			<0.01	a-c	0.01	c<a	④
				c	165	2.55	0.98				b-c	<0.01	c<b	⑤
		40	Identify a key person who can be a bridge to connect with the community to proceed with prior discussion and community development by paying attention not to burden only one person.	a	171	2.96	1.18	30	17.5		a-b	0.46		
				b	185	3.18	0.90			<0.01	a-c	<0.01	c<a	
				c	165	2.60	0.94				b-c	<0.01	c<b	⑤
		41	Go out to the community and positively bring up inquiries in order to receive cooperation from the residents for community development.	a	171	2.80	1.16	29	17.0		a-b	0.13		
				b	185	3.06	0.89			<0.01	a-c	0.16		
				c	165	2.62	1.00				b-c	<0.01	c<b	⑤
		42	Begin with what the residents can do without forcing them, in order to enhance independence of the residents.	a	171	3.27	1.12	18	10.5		a-b	1.00		
				b	185	3.41	0.90			<0.01	a-c	0.01	c<a	
				c	165	3.01	1.03				b-c	<0.01	c<b	
	Utilize policies and systems of national, prefectural and municipal governments.	43	Align the movement of directionality, policies and systems of the national and local governments with the vision targeted by the community.	a	171	3.26	1.00	13	7.6		a-b	0.26		
				b	185	3.11	0.93			<0.01	a-c	<0.01	c<a	
				c	165	2.78	0.98				b-c	0.01	c<b	⑤
		44	Conduct business using the subsidies from national and local governments or the important tasks of the affiliated facility.	a	171	3.12	1.11	17	9.9		a-b	0.03	b<a	
				b	185	2.86	0.95			<0.01	a-c	<0.01	c<a	④
				c	165	2.59	0.97				b-c	0.06		⑤
		45	Exchange information with other municipalities or community general support centers on businesses and methods for community development.	a	171	3.31	1.12	17	9.9		a-b	0.20		
				b	185	3.21	0.98			0.01	a-c	<0.01	c<a	
				c	165	3.01	1.03				b-c	0.15		
	Governmentally act and execute to implement the vision as a business.	46	Can plan a business or project and its content and create a project proposal, budget, etc.	a	171	3.52	0.98	11	6.4		a-b	0.06		
				b	185	3.26	1.06			<0.01	a-c	<0.01	c<a	
				c	165	2.70	1.16				b-c	<0.01	c<b	⑤
		47	Contact supervisors and peers who are not related parties in the administrative area or public health nurses of the affiliated facility to request cooperation to establish a business and obtain unofficial consent.	a	171	3.44	1.04	16	9.4		a-b	0.06		
				b	185	3.21	1.04			<0.01	a-c	<0.01	c<a	
				c	165	2.67	1.10				b-c	<0.01	c<b	⑤
		48	Understand the organization or system of the administration and persistently make a proposal to the administration.	a	171	3.12	1.10	21	12.3		a-b	0.45		
				b	185	3.00	0.89			<0.01	a-c	<0.01	c<a	
				c	165	2.42	1.04				b-c	<0.01	c<b	⑤
		49	Understand and effectively utilize each organization or system of social resources in the community (NPO, social welfare corporation, medical corporation, women's society, etc.)	a	171	3.21	0.93	11	6.4		a-b	0.65		
				b	185	3.13	0.84			<0.01	a-c	<0.01	c<a	
				c	165	2.76	0.92				b-c	<0.01	c<b	⑤
		50	Make use of daily tasks currently engaged in for efforts to solve community issues.	a	171	3.35	0.99	10	5.8		a-b	0.13		
				b	185	3.21	0.82			<0.01	a-c	<0.01	c<a	
				c	165	2.93	0.96				b-c	0.06		⑤
		51	Evaluate businesses implemented and correct them if improvement is necessary.	a	171	3.40	0.94	9	5.3		a-b	0.44		
				b	185	3.31	0.80			<0.01	a-c	<0.01	c<a	
				c	165	3.02	0.97				b-c	0.02	c<b	
		52	Communicate the effect of the business implemented to the residents.	a	171	3.04	1.10	16	9.4		a-b	0.81		
				b	185	2.98	0.85			0.02	a-c	0.02	c<a	④
				c	165	2.77	0.97				b-c	0.28		⑤
Teamwork among three professionals at a CGSC	Collaboratively perform tasks to evenly share them among three job types.	53	Share and collaboratively perform tasks to equally bear burdens.	a	171	3.57	0.78	1	0.6		a-b	0.58		
				b	185	3.64	0.88			0.01	a-c	0.25		
				c	165	3.43	0.84				b-c	0.01	c<b	
		54	Share information among three job types or among peers or other job types by frequent reporting, communication and consultation.	a	171	3.95	0.65	1	0.6		a-b			
				b	185	4.03	0.81			0.16	a-c			③
				c	165	3.88	0.87				b-c			
	Respect the other two job types as professionals to encourage one another.	55	Proactively communicate to develop a relationship that facilitates conversation among three job types or among peers or other job types.	a	171	3.94	0.70	1	0.6		a-b			
				b	185	4.05	0.80			0.18	a-c			③
				c	165	3.97	0.85				b-c			
		56	Listen to the opinion of social workers, chief care managers, peers and other job types without enforcing own opinions.	a	171	4.02	0.58	1	0.6		a-b	0.52		
				b	185	4.09	0.66			0.01	a-c	0.01	a<c	③
				c	165	4.21	0.60				b-c	0.24		
		57	Respect social workers, chief care managers, peers and other job types as professionals.	a	171	4.12	0.62	1	0.6		a-b	0.28		
				b	185	4.21	0.70			<0.01	a-c	<0.01	a<c	③
				c	165	4.39	0.64				b-c	0.16	b<c	
		58	Mutually teach and develop social workers, chief care managers, peers and other job types.	a	171	4.04	0.66	1	0.6		a-b			
				b	185	4.09	0.74			0.62	a-c			③
				c	165	4.02	0.85				b-c			
	Reach out to social workers and chief care managers to obtain understanding for the care prevention business.	59	Approach social workers, chief care managers, peers and other job types to request their cooperation for the duties of public health nurses or for the care prevention business.	a	171	3.75	0.75	3	1.8		a-b			
				b	185	3.90	0.79			0.15	a-c			
				c	165	3.84	0.93				b-c			
		60	Share the community issues with social workers, chief care managers, peers and other job types.	a	171	3.70	0.90	7	4.1		a-b	0.01	a<b	
				b	185	3.97	0.75			0.01	a-c	1.00		
				c	165	3.75	0.95				b-c	0.11		
	Become independent as a public health nurse of a community general support center.	61	Have own opinions as public health nurses among three job types in a community general support center as well as among peers and other job types.	a	171	3.90	0.72	1	0.6		a-b	0.30		
				b	185	4.04	0.69			<0.01	a-c	0.02	c<a	③
				c	165	3.65	0.89				b-c	<0.01	c<b	
		62	Consider what can be done as public health nurses among three job types in a community general support center as well as among peers and other job types, and implement what can be done.	a	171	3.84	0.76	1	0.6		a-b	0.15		
				b	185	4.00	0.75			<0.01	a-c	<0.01	c<a	③
				c	165	3.50	0.94				b-c	<0.01	c<b	
Self-improvement	Keep up with knowledge and experience required to perform duties.	63	Learn by looking at others' support methods to increase one's own ability of discernment.	a	171	3.94	0.63	3	1.8		a-b			
				b	185	4.02	0.47			0.25	a-c			③
				c	165	3.92	0.55				b-c			
		64	Receive advice from supervisors, peers and related parties.	a	171	4.12	0.54	0	0.0		a-b	1.00		
				b	185	4.11	0.51			0.02	a-c	0.01	a<c	③
				c	165	4.27	0.61				b-c	<0.01	b<c	
		65	Pay attention to information on revision of laws, policies and systems to obtain updated information at all times.	a	171	3.66	0.73	0	0.0		a-b	1.00		
				b	185	3.64	0.71			<0.01	a-c	<0.01	c<a	
				c	165	3.33	0.74				b-c	<0.01	c<b	
		66	Proactively attend training to continue self-improvement.	a	171	3.66	0.85	0	0.0		a-b			
				b	185	3.71	0.77			0.52	a-c			
				c	165	3.62	0.81				b-c			
		67	Participate in seminars for public health nurses to expand knowledge on activities of public health nurses in the community.	a	171	3.42	0.90	0	0.0		a-b			
				b	185	3.16	1.03			0.57	a-c			
				c	165	3.25	0.99				b-c			
	Continue to be willing to be involved until the end to perform duties.	68	Persistently make efforts to the end without giving up, keeping tasks in mind at all times.	a	171	3.57	0.77	0	0.0		a-b			
				b	185	3.52	0.76			0.14	a-c			
				c	165	3.44	0.75				b-c			

		69	Make efforts to promptly respond to what can be done if it seems necessary.	a	171	3.84	0.66	0	0.0		a-b			
				b	185	3.82	0.60			0.79	a-c			③
				c	165	3.82	0.70				b-c			
		70	Be responsible to individuals involved and to the community.	a	171	3.96	0.56	0	0.0		a-b			
				b	185	4.05	0.56			0.40	a-c			③
				c	165	3.99	0.65				b-c			
		71	Communicate and explain what cannot be done if it is determined unable to perform with responsibility based on the job volume, role and situation.	a	171	3.70	0.74	1	0.6		a-b			
				b	185	3.66	0.75			0.72	a-c			
				c	165	3.64	0.79				b-c			
		72	Find joy in a job.	a	171	3.65	0.84	0	0.0		a-b	1.00		
				b	185	3.61	0.91			0.03	a-c	0.05		
				c	165	3.39	0.97				b-c	0.83		
		73	Be thankful to supervisors and peers at a workplace.	a	171	4.12	0.67	0	0.0		a-b			
				b	185	4.09	0.65			0.52	a-c			③
				c	165	4.16	0.69				b-c			
	Reflect on own activities as public health nurses at a community general support center.	74	Evaluate own job accomplishments in the past and receive training on what is missing.	a	171	3.48	0.86	0	0.0		a-b			
				b	185	3.39	0.84			0.42	a-c			
				c	165	3.43	0.83				b-c			
		75	Take actions while reflecting on how daily activities lead to the targeted vision.	a	171	3.48	0.81	0	0.0		a-b	0.14		
				b	185	3.31	0.87			0.01	a-c	<0.01	c<a	
				c	165	3.19	0.84				b-c	0.54		
Job management	Perform duties in consideration of the job schedule and priority in order to provide effective support at all times.	76	Manage a schedule and organize tasks to perform duties in an orderly way.	a	171	3.65	0.71	0	0.0		a-b			
				b	185	3.76	0.73			0.33	a-c			
				c	165	3.67	0.75				b-c			
		77	Perform in consideration of priority when working on multiple cases and duties.	a	171	3.86	0.63	0	0.0		a-b	0.05		
				b	185	4.02	0.63			0.02	a-c	1.00		③
				c	165	3.85	0.70				b-c	0.06		
		78	When emergency response is required, promptly determine what to do with the current duties.	a	171	3.77	0.66	0	0.0		a-b	0.13		
				b	185	3.91	0.64			<0.01	a-c	0.02	c<a	
				c	165	3.52	0.79				b-c	<0.01	c<b	
		79	Work in consideration of the balance between individual support and tasks for community development.	a	171	3.37	0.98	10	5.8		a-b	1.00		
				b	185	3.39	0.77			<0.01	a-c	0.01	c<a	
				c	165	3.08	0.85				b-c	<0.01	c<b	

a: PA public health nurse b: CGSC experienced public health nurse c: CGSC newcomer public health nurse.

d: Number and percentage of people who responded with “0: No opportunity to practice it”, which was an option only for PA public health nurses

e: Kruskal-Wallis test, f: Donn test

g: The circled numbers in the judgment indicate (2): competency acquired through certain amount of experience at CGSC, (3) common competency, (4) competency that was not yet sufficiently acquired even by CGSC experienced public health nurses, and (5) competency lacking in CGSC newcomer public health nurses.
